# Genome Sequence of Enterovirus D68 from St. Louis, Missouri, USA

**DOI:** 10.3201/eid2101.141605

**Published:** 2015-01

**Authors:** Kristine M. Wylie, Todd N. Wylie, Anthony Orvedahl, Richard S. Buller, Brandi N. Herter, Vincent Magrini, Richard K. Wilson, Gregory A. Storch

**Affiliations:** Washington University School of Medicine, St. Louis, Missouri, USA

**Keywords:** enterovirus D68, enterovirus, genomic, outbreak, respiratory, asthma, pediatric, viruses, St. Louis

**To the Editor:** During the current (2014) enterovirus/rhinovirus season in the United States, enterovirus D68 (EV-D68) is circulating at an unprecedented level. As of October 6, 2014, the Centers for Disease Control and Prevention (CDC) had confirmed 594 cases of EV-D68 infection in 43 states and the District of Columbia (http://www.cdc.gov/non-polio-enterovirus/outbreaks/EV-D68-outbreaks.html); the actual number of cases was undoubtedly much higher. In mid-August, hospitals in Missouri and Illinois noticed an increased number of patients with severe respiratory illness (*1*). We observed this pattern at St. Louis Children’s Hospital in St. Louis, Missouri.

Resources for studying this virus are limited. Before the current season, only 7 whole-genome sequences and 5 additional complete coding sequences of the virus were available. Therefore, determining whether there are genomic elements associated with rapid spread or severe and unusual disease was not possible. 

To address these limitations, we determined the complete coding sequence of 1 strain from St. Louis by using high-throughput sequencing of nucleic acid from a clinical sample. To evaluate the sequence diversity in EV-D68 strains circulating in the St. Louis metropolitan area, we also generated partial-genome sequences from 8 more EV-D68–positive clinical samples from St. Louis. During the preparation of this article, CDC generated and submitted to GenBank 7 complete or nearly complete genome sequences from viruses obtained from the Midwest. We documented the diversity of the sequences of strains from St. Louis and compared them to publicly available sequences.

The methods are described in brief here and in more detail in the Technical Appendix. This study was conducted under a protocol approved by the Human Research Protection Office of Washington University in St. Louis. 

Patients were categorized retrospectively as having mild, moderate, or severe disease if they had been discharged home from the emergency unit, admitted to general wards, or admitted to the pediatric intensive care unit, respectively. Residual material from a subset of nasopharyngeal specimens positive for rhinovirus/enterovirus (tested by the BioFire FilmArray Respiratory Panel [BioFire Diagnostics, Salt Lake City, UT, USA] at the Clinical Virology Laboratory, St. Louis Children’s Hospital) was selected for high-throughput sequencing. Total nucleic acid was extracted from clinical samples by using NucliSENS easyMAG (bioMérieux, Marcy l'Etoile, France) and used to make dual-indexed sequencing libraries. Enterovirus/rhinovirus sequences were enriched by using a NimbleGen custom sequence capture reagent (Roche/NimbleGen, Madison, WI, USA), which as of February 2014 was selective for all complete enterovirus and rhinovirus genomes in GenBank. Sequence data were generated on an Illumina HiSeq 2500 (Illumina Inc., San Diego, CA, USA). Sequences were assembled with IDBA-UD (*2*) and manually improved. The most contiguous genome was annotated by using VIGOR (*3*). Publicly available sequences were downloaded and compared by using the National Institute of Allergy and Infectious Diseases Virus Pathogen Resource (http://www.viprbrc.org) (*4*). Variants were identified by using VarScan (*5*). The sequence was deposited in GenBank under accession no. KM881710, BioProject PRJNA263037.

For 14 of the 17 samples, high-throughput sequencing data were interpretable (Technical Appendix Table); for the other 3 samples, the number of virus sequence reads was too low to distinguish them from sample cross-talk, which occurs during high-throughput sequencing analysis (*6*). Of the 14 typed samples, EV-D68 sequences were detected in 7 of 10 samples from patients with severe disease, 2 of 2 with moderate disease, and 0 of 2 with mild disease. The complete coding sequence was assembled from sample EV-D68_STL_2014_12. The most closely related genomes from previous seasons were Thailand, CU134, and CU171 (*7*) (Figure, panel A). Several of the genome sequences obtained from Missouri strains from this season, which had been sequenced by CDC, were very similar to this genome sequence. Comparison of the virus protein 1 sequence with that of publicly available sequences indicated that the strain from St. Louis and the strain from Missouri (CDC) cluster with virus strains identified in Europe and Asia within the past several years (Figure, panel B). The St. Louis virus shared 97%–99% aa sequence identity with all other sequenced strains. We observed little variation in the strains from St. Louis because they shared 98%–99% nt sequence identity (Technical Appendix Figure).

**Figure:**
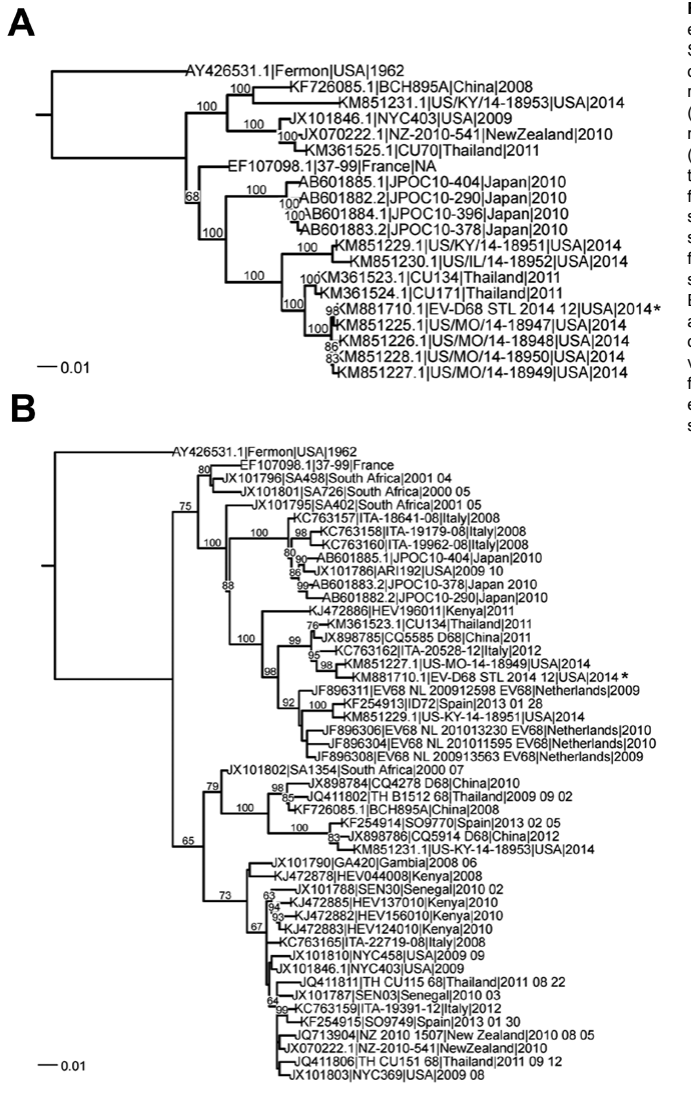
Phylogenetic comparison of enterovirus D68 (EV-D68) obtained from St. Louis, Missouri, USA, in 2014, with other sequenced strains. The phylogenetic relationships of genome sequences (nucleotides) were estimated by using the maximum-likelihood method with RAxML (http://www.viprbrc.org) bootstrapped 100 times. A) Comparison of genome sequences for full-length and nearly full-length strains. B) Comparison of virus protein 1 sequences. Sequences were downloaded from ViPR (http://www.viprbrc.org) and supplemented with strains from the 2014 EV-D68 season. Sequences were clustered at 99% identity to obtain a representative set of sequences, which are shown. Bootstrap values are indicated on each tree. The strain from St. Louis is indicated by an asterisk on each tree. Scale bars indicate nucleotide substitutions per site.

We provide a genome sequence from the 2014 outbreak of EV-D68 infection in St. Louis, Missouri. This sequence seems to be highly representative of the strains circulating in St. Louis during this time because the other genomes we partially sequenced are very similar. To our knowledge, no amino acids have been associated with virulence or increased infectivity of EV-D68; therefore, we cannot associate the changes we observed in these genomes to phenotypic traits. Because changes in the 5′ untranslated region have the potential to affect the rate of replication (*8*–*10*), it is possible that minor genome changes are responsible for the rapid spread and high severity of disease in 2014. Correlation between clinical features of patients in conjunction with additional genomic analysis might provide further insight into the pathogenetic determinants of this strain. Therefore the genome sequence of EV-D68 determined from the 2014 outbreak in St. Louis, Missouri, provides a resource for tracking and genomic comparison of this rapidly spreading virus.

Technical Appendix Supplemental methods.

## References

[1] Midgley CM, Jackson MA, Selvarangan R, Turabelidze G, Obringer E, Johnson D, Severe respiratory illness associated with enterovirus D68— Missouri and Illinois, 2014. MMWR Morb Mortal Wkly Rep. 2014;63:798–9 . 25211545 PMC4584696

[2] Peng Y, Leung HCM, Yiu SM, Chin YL. IDBA-UD: a de novo assembler for single-cell and metagenomic sequencing data with highly uneven depth. Bioinformatics. 2012;28:1420–8. **PMID: 22495754** 10.1093/bioinformatics/bts174 22495754

[3] Wang S, Sundaram JP, Stockwell TB. VIGOR, an annotation program for small viral genomes. Bioinformatics. 2010;11:451 . 10.1186/1471-2105-11-451 20822531 PMC2942859

[4] Pickett BE, Sadat EL, Zhang Y, Noronha JM, Squires RB, Hunt V, ViPR: an open bioinformatics database and analysis resource for virology research. Nucleic Acids Res. 2012;40:D593–8 . 10.1093/nar/gkr859 22006842 PMC3245011

[5] Koboldt DC, Zhang Q, Larson DE, Shen D, McLellan MD, Lin L, VarScan 2: somatic mutation and copy number alteration discovery in cancer by exome sequencing. Genome Res. 2012;22:568–76 . 10.1101/gr.129684.111 22300766 PMC3290792

[6] Kircher M, Sawyer S, Meyer M. Double indexing overcomes inaccuracies in multiplex sequencing on the Illumina platform. Nucleic Acids Res. 2012;40:e3 . 10.1093/nar/gkr771 22021376 PMC3245947

[7] Prachayangprecha S, Schapendonk CM, Koopmans MP, Osterhaus AD, Schürch AC, Pas SD, Exploring the potential of next-generation sequencing in detection of respiratory viruses. J Clin Microbiol. 2014;52:3722–30 . 10.1128/JCM.01641-14 25100822 PMC4187785

[8] Yeh MT, Wang SW, Yu CK, Lin KH, Lei HY, Su IJ, A single nucleotide in stem loop II of 5′-untranslated region contributes to virulence of enterovirus 71 in mice. PLoS ONE. 2011;6:e27082 . 10.1371/journal.pone.0027082 22069490 PMC3206083

[9] Chang SC, Li WC, Chen GW, Tsao KC, Huang CG, Huang YC, Genetic characterization of enterovirus 71 isolated from patients with severe disease by comparative analysis of complete genomes. J Med Virol. 2012;84:931–9 . 10.1002/jmv.23287 22499017

[10] M'hadheb-Gharbi MB, Kean KM, Gharbi J. Molecular analysis of the role of IRES stem-loop V in replicative capacities and translation efficiencies of coxsackievirus B3 mutants. Mol Biol Rep. 2009;36:255–62. 10.1007/s11033-007-9174-3 18027104

