# Supplementary material for: Genome Sequence of Enterovirus D68 from St. Louis, Missouri, USA
Source: Emerg Infect Dis. 2015 Jan;21(1):184–6. doi: 10.3201/eid2101.141605 (PMC4285240; doi:10.3201/eid2101.141605)
Supplement: Technical Appendix — Supplemental methods. [file 14-1605-Techapp-s1.pdf]

Article DOI: http://dx.doi.org/10.3201/eid2101.141605

# Genomic Characterization of Enterovirus D68 from St. Louis, Missouri, USA

## Technical Appendix

## Supplemental Methods

**Genome sequencing**: Total nucleic acid was extracted using the NucliSENS easyMAG instrument (bioMérieux, Marcy l'Etoile, France). DNase-treated samples were used to make cDNA as previously described (*1*,*2*). Dual-indexed sequencing libraries were constructed. Enterovirus/rhinovirus sequences were enriched by using a NimbleGen custom sequence capture reagent (Roche/NimbleGen, Madison, WI, USA) with probes targeting all complete enterovirus and rhinovirus genomes in GenBank as of February 2014. Paired-end, 100-base sequences were generated on the Illumina HiSeq 2500 platform in rapid mode (Illumina Inc., San Diego, CA, USA). Primer sequences were removed with Flexbar (*3*). Sequences were assembled with IDBA-UD (*4*). Data were visualized by using Tablet (*5*). The most contiguous sequence was manually evaluated and improved. The genome was annotated with VIGOR (*6*,*7*). Genome and VP1 sequences were compared by using the NIAID Virus Pathogen Resource (ViPR) (http://www.viprbrc.org) (*8*). In brief, VP1 sequences were downloaded from ViPR, and unique sequences that spanned a 723 base pair amplicon that was commonly deposited in the database were used (positions 2209–2931 in the St. Louis strain genome). Newly sequenced CDC strains were added to the datasets. VP1 sequences were clustered at 99% identity to obtain an easily visualized set of sequences. Nucleotide sequences were aligned with MUSCLE (*9*) and compared by the maximum-likelihood method by using RAxML (*10*) bootstrapped 100 times. Trees were visualized by using iTOL (*11*). Viruses were typed by aligning sequence reads to a complete set of viral reference genomes using BWA mem (*12*). Variants were identified by using VarScan (*13*). The sequence of the St.

Louis reference strain was deposited into GenBank, accession no. KM881710, BioProject PRJNA263037.

**References**

1. Wang D, Coscoy L, Zylberberg M, Avila PC, Boushey HA, Ganem D, et al. Microarray-based detection and genotyping of viral pathogens. Proc Natl Acad Sci USA. 2002;99:15687–92. http://dx.doi.org/10.1073/pnas.242579699

2. Wang D, Urisman A, Liu Y-T, Springer M, Ksiazek TG, Erdman DD, et al. Viral discovery and sequence recovery using DNA microarrays. PLoS Biol. 2003;1:E2.

3. Dodt M, Roehr JT, Ahmed R, Dieterich C. FLEXBAR-Flexible Barcode and Adapter Processing for Next-Generation Sequencing Platforms. Biology (Basel); 2012;1:895–905.

4. Peng Y, Leung, HCM, Yiu SM, and Chin YL. IDBA-UD: a de novo assembler for single-cell and metagenomic sequencing data with highly uneven depth. Bioinformatics. 2012;28:1420–8.

5. Milne I, Stephen G, Bayer M, Cock PJA, Pritchard L, Cardle L, et al. Using Tablet for visual exploration of second-generation sequencing data. Brief Bioinform. 2013;14:193–202.

6. Wang S, Sundaram JP, Spiro D. VIGOR, an annotation program for small viral genomes. BMC Bioinformatics. 2010;11:451. PubMed

7. Wang S, Sundaram JP, Stockwell TB. VIGOR extended to annotate genomes for additional 12 different viruses. Nucleic Acids Res. 2012;40:W186–92.

8. Pickett BE, Sadat EL, Zhang Y, Noronha JM, Squires RB, Hunt V, et al. ViPR: an open bioinformatics database and analysis resource for virology research. Nucleic Acids Res. 2012;40:D593–8.

9. Edgar RC. MUSCLE: a multiple sequence alignment method with reduced time and space complexity. Bioinformatics. 2004;5:113.

10. Stamatakis A. RAxML-VI-HPC: maximum likelihood-based phylogenetic analyses with thousands of taxa and mixed models. Bioinformatics. 2006;22:2688–90.

11. Letunic I, Bork P. Interactive Tree Of Life v2: online annotation and display of phylogenetic trees made easy. Nucleic Acids Res. 2011;39:W475–8.

12. Li H, Durbin R. Fast and accurate short read alignment with Burrows-Wheeler transform. Bioinformatics. 2009;25:1754–60.

13. Koboldt DC, Zhang Q, Larson DE, Shen D, McLellan MD, Lin L, et al. VarScan 2: somatic mutation and copy number alteration discovery in cancer by exome sequencing. Genome Res. 2012;22:568–76. PubMed

Technical Appendix Table. Disease severity and virus type

| Sample | Severity | Virus typing results |
|---|---|---|
| EV-D68_STL_2014_1 | Severe | EV-D68 |
| EV-D68_STL_2014_2 | Severe | Rhinovirus C |
| EV-D68_STL_2014_3 | Severe | EV-D68 |
| EV-D68_STL_2014_4 | Severe | Rhinovirus B |
| EV-D68_STL_2014_5 | Severe | EV-D68 |
| EV-D68_STL_2014_6 | Severe | EV-D68 |
| EV-D68_STL_2014_7 | Severe | EV-D68 |
| EV-D68_STL_2014_8 | Severe | Rhinovirus C |
| EV-D68_STL_2014_9 | Severe | EV-D68 |
| EV-D68_STL_2014_10 | Severe | EV-D68 |
| EV-D68_STL_2014_11 | Moderate | Not determined |
| EV-D68_STL_2014_12 | Moderate | EV-D68 |
| EV-D68_STL_2014_13 | Moderate | EV-D68 |
| EV-D68_STL_2014_14 | Mild | Rhinoviruses A, C, and coxsackievirus |
| EV-D68_STL_2014_15 | Mild | Not determined |
| EV-D68_STL_2014_16 | Mild | Rhinovirus A |
| EV-D68_STL_2014_17 | Mild | Not determined |

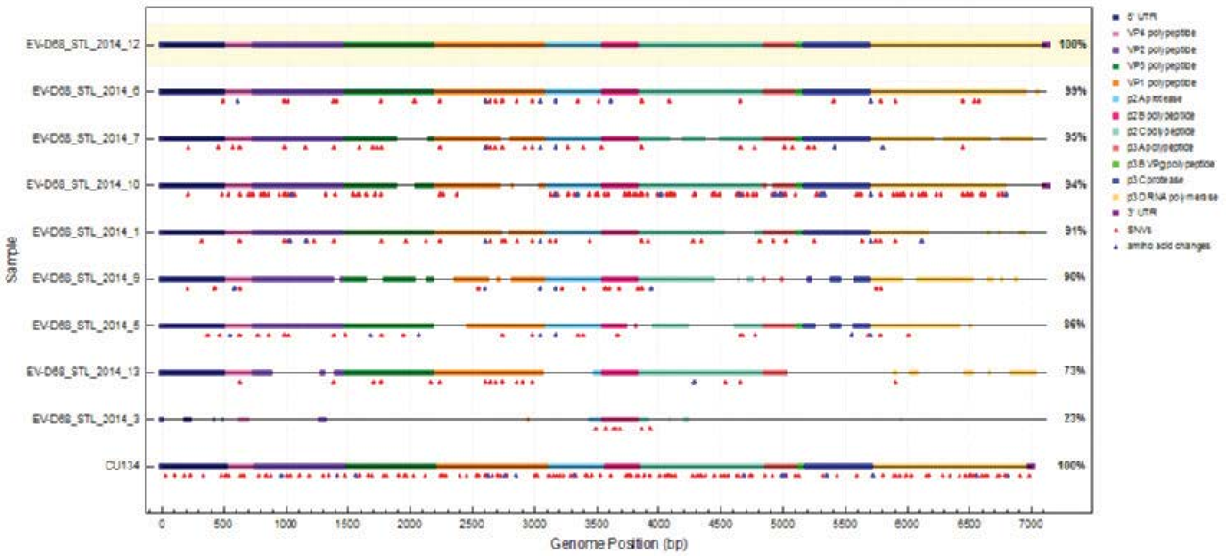

**Appendix Figure.** Genomic comparison of St. Louis EV-D68 strains. Genomic differences between the 9 St. Louis strains and the most closely related sequence (CU134) from the public database are illustrated. Each genome is represented by a horizontal line. The genome positions (x-axis) are given relative to the St. Louis EV-D68_STL_2014_12 strain, which is the top genome in the figure. The genome features are indicated by colored bars across the genome and are described in detail in the key to the right of the figure. Genome features that are not represented in the sequence data from the sample (i.e., not covered by reads) are indicated by a thin black line and no colored bar. Total percentages of the genome that is represented in the sequencing data are shown to the right of each genome representation. Single nucleotide changes, compared with EV-D68_STL_2014_12, are shown by red triangles. Amino acid changes are indicated by blue triangles.
